# Analysis of single umbilical artery with concurrent congenital anomaly: Is it a risk factor for poor prognosis? A cross-sectional study

**DOI:** 10.18502/ijrm.v22i2.15710

**Published:** 2024-03-25

**Authors:** Na Hyun Lee, Hee Joung Choi

**Affiliations:** Department of Pediatrics, Keimyung University School of Medicine, Daegu, Republic of Korea.

**Keywords:** Single umbilical artery, Congenital abnormalities, Perinatal mortality.

## Abstract

**Background:**

A single umbilical artery (SUA) may coexist with a single anomaly or multiple congenital anomalies. Although anomalies associated with SUA can primarily cause high perinatal mortality, their clinical significance has not been evaluated.

**Objective:**

We investigated the relationship between the clinical features and the type or number of concurrent anomalies in neonates with SUA.

**Materials and Methods:**

In this cross-sectional study, 104 neonates with SUA were enrolled from January 2000 to December 2020 at Dongsan hospital, Daegu, South Korea. Data on the maternal history and the neonates demographic characteristics, clinical course, chromosomal analysis, and congenital anomalies, were collected.

**Results:**

Among the neonates with SUA included, 77 (74.0%) had one or more congenital anomalies; 66 (63.5%) were cardiac, 20 (19.2%) were genitourinary, 12 (11.5%) were gastrointestinal, 5 (4.8%) were central nervous system, 12 (11.5%) were skeletal, and 5 (4.8%) were facial anomalies. The number of concurrent anomalies ranged from 0–4. Neonates with SUA and concurrent gastrointestinal anomaly had a high incidence of initial positive ventilation, intubation, and inotropic drug use and lower Apgar score at 1 min and 5 min. 7 (6.7%) neonates with SUA died. Low birth weight (odds ratio = 6.16, p = 0.05), maternal multiparity (2.41, p = 0.13), gastrointestinal anomaly (5.06, p = 0.11), and initial cardiac resuscitation (7.77, p = 0.11) were risk factors for mortality in neonates with SUA.

**Conclusion:**

Neonates with SUA and concurrent gastrointestinal anomaly, low birth weight, maternal multiparity, and initial cardiac resuscitation had poor outcomes.

## 1. Introduction

The umbilical cord consists typically of one vein and 2 arteries, and abnormalities in the umbilical cord are associated with fetal death and congenital anomalies. A single umbilical artery (SUA), characterized by the absence of one of the umbilical arteries, is one of the most common fetal abnormalities identified during prenatal screening. Its incidence ranges from 0.3–3.5% in liveborn neonates and is much higher in abortuses and during autopsies (2.4–7%) (1, 2).

Fetuses with SUA have many comorbidities during the prenatal, antenatal, and postnatal periods. Fetuses with SUA have a high incidence of fetal hydrops and death (53%) (2). Compared to neonates with a 3-vessel cord, neonates with SUA were more likely associated with complications, such as intrauterine fetal growth restriction, polyhydramnios or oligohydramnios, preterm delivery, low birth weight, delivery by cesarean section owing to fetal distress, low Apgar score, perinatal death, admission to the neonatal intensive care unit, and placental abnormalities (placental abruption, placenta previa, or cord prolapse) (3–5).

In approximately 65–80% of neonates with SUAs, SUA may be the only finding without the presence of any structural abnormalities (isolated SUA) (6, 7). However, one-third of neonates with SUA have concurrent congenital malformations. Many studies have analyzed the frequency and types of congenital defects associated with SUA; however, the findings have not been consistent. The incidence of fetal malformations associated with SUA is 13–50% (8), which is increased to 66.3% in cases of fetal deaths (9). In most cases, malformations involve the genitourinary, cardiovascular, gastrointestinal, musculoskeletal, and central nervous systems (CNS), and SUA may coexist with a single or multiple defects (6, 8). Neonates with SUA have a 6.77 times greater risk of congenital anomalies than in neonates with a 3-vessel cord (10). Moreover, the incidence of fetuses with SUA and concurrent multiple malformations varies from 7.4–72% (11, 12).

Although malformations associated with SUA can primarily cause high perinatal mortality, their clinical significance has not been well evaluated, especially in neonates with multiple malformations (6). We believe that morbidity and mortality rates in neonates with SUA may differ according to the type or number of associated congenital defects.

This study aimed to investigate the incidences and types of congenital anomalies in neonates with SUA. Furthermore, we assessed the clinical features according to the type and number of concurrent congenital anomalies in neonates with SUA.

## 2. Materials and Methods 

### Study design

In this cross-sectional study, we reviewed the medical records of neonates who were admitted to the neonatal intensive care unit of Dongsan hospital, Daegu, South Korea between January 2000 and December 2020. We enrolled 104 neonates who were confirmed to have an SUA by examination of the umbilicus soon after birth. Neonates who did not undergo a complete investigation to detect congenital anomalies were excluded. The type and number of combined congenital anomalies categorized the neonates with SUA. According to the types of congenital anomalies, they were grouped into 6 categories; cardiac, gastrointestinal, genitourinary, CNS, facial, and skeletal anomalies. Based on the 2D-echocardiography results in the medical records, any isolated patent foramen ovale or patent ductus arteriosus within 2 wk after birth was not included as cardiac anomalies. According to the number of concurrent congenital anomalies, they were grouped ranging from 0–4. Additionally, neonates who had 3 or more vertebral defects, anal atresia, cardiac defects, tracheoesophageal fistula, renal anomalies, and limb (VACTERL) abnormalities were assigned to the VACTERL association (13).

To analyze the clinical features, the collected data included sex, gestational age, birth weight, birth height, maternal history (maternal age, parity, diabetes mellitus, hypertension, amount of amniotic fluid, premature rupture of membrane, chorioamnionitis, and steroid medication), birth history (Apgar score at 1 and 5 min and whether resuscitation was required immediately after birth), clinical course (respiratory distress syndrome, air leak syndrome, pulmonary hemorrhage, pulmonary hypertension, intraventricular hemorrhage, necrotizing enterocolitis, acute renal failure, infection, retinopathy of prematurity, hearing impairment, respiratory support, inotropic drug use, and parenteral nutrition and hospitalization duration), and mortality. We compared the clinical features of neonates with and without each type of congenital anomalies, and the numbers of concurrent congenital anomalies. Furthermore, we compared the clinical features and combined anomalies depending on the death as a clinical outcome.

### Ethical considerations 

This study was approved by the Institutional Review Board of Keimyung University Dongsan hospital, Daegu, Korea (Code: 2022-05-027) and was conducted in accordance with the Declaration of Helsinki and its later amendments.

### Statistical analysis

For the continuous data, the mean differences between the 2 groups were compared using an independent *t* test, whereas for the categorical data, the proportion of the differences between the 2 groups were compared using the Chi-square test. To determine the risk factors for postnatal mortality, we performed the firth's logistic regression model and receiver operating characteristic curve analysis. Since the infant mortality rate was low, the Fisher's logistic regression model was used. This model is suitable for handling rare events (14). The stepwise method was used to select variables for the multiple logistic regression analysis, and the significance level was set to 0.15. The statistical analysis was performed using the SAS 9.4 (Copyright (c) 2002–2012, SAS Institute Inc., Cary, NC, USA), and the significance level was set to 5%.

## 3. Results 

### Study characteristics and associated anomalies

**Figure 1:**
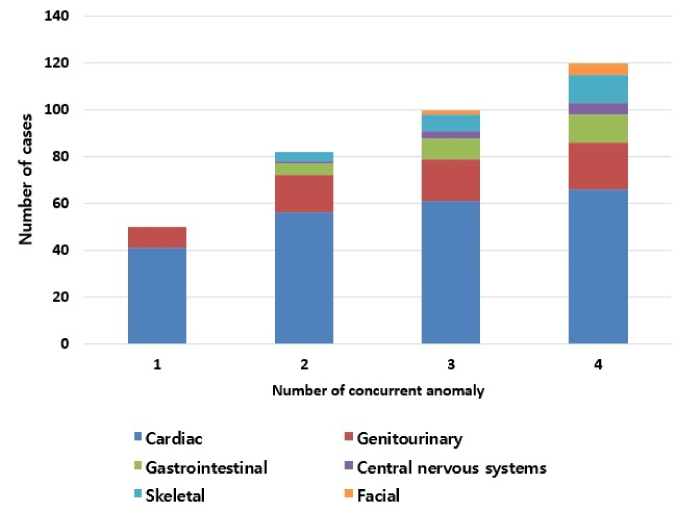
The cumulative number of cases in 6 types of congenital anomalies according to the total number of concurrent anomalies.

A total of 104 neonates (45 boys and 59 girls) were enrolled, and 77 (74.0%) had 1 or more congenital anomalies. The number of cases in each anomaly type were: 66 (63.5%) in cardiac, 20 (19.2%) in genitourinary, 12 (11.5%) in gastrointestinal, 5 (4.8%) in CNS, 12 (11.5%) in skeletal, and 5 (4.8%) in facial anomalies. Figure 1 shows the number of neonates with SUA who have concurrent anomalies; 27 neonates had no concurrent anomaly, 50 had 1 anomaly, 16 had 2 anomalies, 6 had 3 anomalies, and 5 had 4 anomalies.

Cardiac anomalies included a ventricular septal defect, atrial septal defect, patent ductus arteriosus, pulmonary atresia, double outlet right ventricle, aortic arch anomaly, and persistent left superior vena cava. The genitourinary anomalies were renal agenesis/hypoplasia, ectopic kidney, horseshoe kidney, hydronephrosis, hydroureter, vesicourethral reflux, and double collecting system. The gastrointestinal anomalies were tracheoesophageal fistula with or without esophageal atresia, imperforate anus, duodenal atresia, and Bochdalek hernia. CNS anomalies included microcephaly, ventriculomegaly, absent septum pellucidum, and multiple ependymal cysts. Skeletal anomalies were butterfly vertebrae, hemivertebra, polydactyly, thumb hypoplasia, lipomyelomeningocele, spinal cord tethering, clubfoot, congenital hip dislocation, and pes planus. Facial anomalies were brachycephaly, macrotia, facial palsy, facial asymmetry, and low-set ears. We also found other anomalies such as hearing impairment, tongue tie, simian line, situs inversus, and cryptorchidism.

5 patients (4.5%) were associated with VACTERL. Neonates with SUA and concurrent VACTERL had a higher incidence of abnormal hearing test results (p= 0.004) and longer duration of parenteral nutrition (p= 0.03) than those of neonates with SUA but without VACTERL association. No significant difference was observed in the mortality rate between neonates associated with VACTERL and those without VACTERL association.

### Clinical findings according to the combined congenital anomalies

Based on the 6 categories of combined congenital anomalies, the clinical features were compared between neonates with and without anomalies (Table I). Depending on the type of anomalies, there were various clinical distinctions. Neonates with SUA and concurrent cardiac anomalies (n = 66) had significantly higher incidence of oligohydramnios, apnea, and chromosomal analysis, and significantly longer duration of incubator care, parenteral nutrition, and hospitalization than in those without concurrent cardiac anomalies (n = 38). No significant difference was observed between the neonates with SUA with (n = 20) and without (n = 84) concurrent genitourinary anomalies. Neonates with SUA and concurrent gastrointestinal anomalies (n = 12) had significantly higher incidence of skeletal anomalies, oligohydramnios, polyhydramnios, initial positive ventilation, intubation, and inotropic drug use, significantly higher Apgar scores at 1 min and 5 min, and significantly longer duration of hospitalization than in those without gastrointestinal anomalies (n = 92). Neonates with SUA and concurrent CNS anomalies (n = 5) had significantly higher incidence of facial anomalies, initial positive ventilation, intubation, and pulmonary hemorrhage than in those without CNS anomalies (n-99). Neonates with SUA and concurrent skeletal anomalies (n = 12) had had significantly higher incidence of gastrointestinal anomalies, facial anomalies, and oligohydramnios than in those without skeletal anomalies (n = 92). Neonates with SUA and concurrent facial anomalies (n = 5) had significantly higher incidence of CNS anomalies, skeletal anomalies, chromosomal analysis, and pulmonary hemorrhage than in those without facial anomalies (n = 99).

Based on the number of concurrent congenital anomalies, the correlation analysis showed a significantly positive relationship between the number of associated anomalies and the duration of incubator care (Spearman's rho = 0.264, p= 0.01), parenteral nutrition (Spearman's rho = 0.254, p= 0.01), and hospitalization (Spearman's rho = 0.189, p = 0.05). It could be seen that the greater the number of accompanying anomalies, the longer the incubator care, parenteral nutrition, and hospitalization duration.

### The clinical findings and risk factors according to mortality

Of the 104 neonates, 7 (6.7%) died. Neonates with SUA in the death group had a significantly lower birth weight (p= 0.03), lower Apgar scores at 1 ${\;}^{\text{st}}$ min (p= 0.02) and 5 ${\;}^{\text{th}}$ min (p= 0.03), higher incidences of low birth weight (p= 0.04), initial cardiac resuscitation (p= 0.001), initial positive ventilation (p < 0.001), initial intubation (p < 0.001), respiratory distress syndrome (p < 0.001), air leak syndrome (p= 0.002), pulmonary hypertension (p= 0.004), and inotropic drug use (p < 0.001), and longer duration of invasive ventilator support (p= 0.02) than those of neonates in the alive group (Table II).

**Figure 2:**
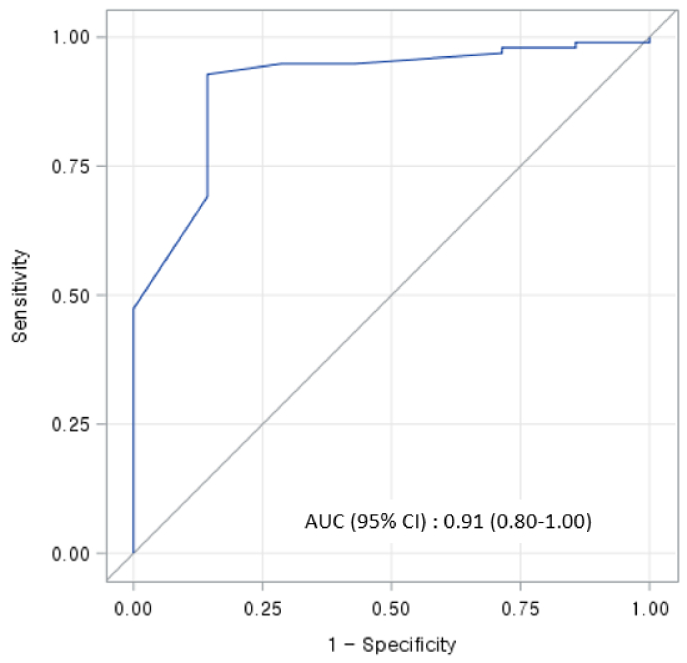
Receiver operator characteristics curves analysis for the risk of the postnatal mortality in neonates with SUA, including low birth weight, maternal multiparity, gastrointestinal anomaly, and initial cardiac resuscitation. AUC: Area under the curve, CI: Confidence intervals.

Using the firth's logistic regression model, we found that mortality was associated with low birth weight (OR = 6.16, p= 0.05), maternal multiparity (OR = 2.41, p= 0.13), gastrointestinal anomaly (OR = 5.06, p= 0.11), and initial cardiac resuscitation (OR = 7.77, p= 0.02) (Table III). A receiver operator characteristics analysis for the risk of the postnatal mortality in neonates with SUA, including low birth weight, maternal multiparity, gastrointestinal anomaly, and initial cardiac resuscitation is shown in figure 2. According to the logistic regression model, the death prediction probability was 90.6%.

### Genetic surveillance

A chromosomal analysis was frequently conducted when neonates with SUA had cardiac or facial anomalies. Of the 22 neonates who underwent chromosomal analysis, 4 had the following abnormal results: 47,XX,t(1:18)(p:33;q10),+18, 46,XX,del(5)(p14),add(20)(p12), 46,X,i(Xq10), arr 2q33,3q37.3(204,91,425–242,75,910).

**Table 1:** Clinical features with significant differences according to the presence of each type of congenital anomalies in neonates with SUA

**Anomaly type**		**Clinical features**	**With anomaly**	**Without anomaly**	**P** ***-*** **value**
**Cardiac anomaly **		Number	66	38	
	Oligohydramnios*		11 (16.7)	0 (0.0)	0.02
	Apnea*		18 (27.3)	3 (7.9)	0.03
	Incubator care day**		15.6 ± 2.2	6.6 ± 1.1	0.01
	Parenteral nutrition day**		7.4 ± 1.4	2.9 ± 0.9	0.05
	Hospitalization day**		22.8 ± 2.6	11.6 ± 1.3	0.01
	Chromosomal analysis*		17 (25.8)	3 (7.9)	0.05
**Genitourinary anomaly**		Number	20	84	
**Gastrointestinal anomaly**		Number	12	92	
	Skeletal anomaly*		4 (33.3)	8 (8.7)	0.04
	Oligohydramnios*		4 (33.3)	7 (7.6)	0.03
	Polyhydramnios*		3 (25.0)	3 (3.3)	0.02
	Initial positive ventilation*		8 (66.7)	16 (17.4)	0.001
	Initial intubation*		5 (41.7)	10 (10.9)	0.02
	Apgar score at 1 min**		4.8 ± 2.6	6.8 ± 1.7	0.02
	Apgar score at 5 min**		6.8 ± 2.3	8.2 ± 1.3	0.05
	Inotropic drug use*		5 (41.7)	11 (12.0)	0.02
	Hospitalization day**		33.6 ± 3.0	16.8 ± 2.1	0.02
**Central nervous system anomaly**		Number	5	99	
	Facial anomaly*		2 (40.0)	3 (3.0)	0.01
	Initial positive ventilation*		4 (80.0)	20 (20.2)	0.01
	Initial intubation*		3 (60.0)	12 (12.1)	0.02
	Pulmonary hemorrhage*		1 (20.0)	0 (0.0)	0.03
**Skeletal anomaly**		Number	12	92	
	Gastrointestinal anomaly*		4 (33.3)	8 (8.7)	0.04
	Facial anomaly*		5 (41.7)	0 (0.0)	< 0.001
	Oligohydramnios*		4 (33.3)	7 (7.6)	0.03
**Facial anomaly**		Number	5	99	
	Central nervous system anomaly*		2 (40.0)	3 (3.0)	0.01
	Skeletal anomaly*		5 (100.0)	7 (7.1)	< 0.001
	Chromosomal analysis*		4 (80.0)	16 (16.2)	0.003
	Pulmonary hemorrhage*		1 (20.0)	0 (0.0)	0.03
*Data presented as number (%), Chi-square test. **Data presented as Mean ± SD, independent *t* test. SUA: Single umbilical artery					

**Table 2:** Comparison of maternal and fetal features in neonates with SUA according to pregnancy outcomes

**Features/Outcomes**	**Alive (n = 97)**	**Death (n = 7)** * *
Multiparity (> 2)*	9 (9.3)	2 (28.6)
Low birth weight (< 2500 gr)*	9 (9.3)	3 (42.9)
Birth weight (gr)**	2411.1 ± 721.7	1775.7 ± 717.2
1 ${\;}^{\text{st}}$ min Apgar**	6.8 ± 1.5	3.3 ± 2.9
5 ${\;}^{\text{th}}$ min Apgar**	8.3 ± 1.2	5.1 ± 2.8
Initial cardiac resuscitation*	8 (8.3)	4 (57.1)
Initial positive ventilation*	18 (18.6)	6 (85.7)
Initial intubation*	9 (9.3)	6 (85.7)
Respiratory distress syndrome*	18 (18.6)	6 (85.7)
Air leak syndrome*	2 (2.1)	3 (42.9)
Pulmonary hypertension*	5 (5.2)	3 (42.7)
Duration of invasive ventilation**	1.7 ± 0.5	6.3 ± 0.7
Inotropic drug use*	9 (9.3)	7 (100.0)
Cardiac anomaly*	62 (63.9)	4 (57.1)
Genitourinary anomaly*	19 (19.6)	1 (14.3)
Gastrointestinal anomaly*	10 (10.3)	2 (28.6)
Central nervous systems anomaly*	4 (4.1)	1 (14.3)
Skeletal anomaly*	11 (11.3)	1 (14.3)
Facial anomaly*	4 (4.1)	1 (14.3)
*Data presented as number (%). **Data presented as Mean ± SD. SUA: Single umbilical artery		

**Table 3:** Logistic regression analysis for the risk of mortality in neonates with SUA

**Variables**	**Odds ratios**	**95% confidence intervals**	**P** ***-*** **value**
**Low birth weight **	6.16	1.00–38.03	0.05
**Maternal multiparity**	2.41	0.78–7.38	0.13
**Gastrointestinal anomaly**	5.06	0.71–36.24	0.11
**Initial cardiac resuscitation**	7.77	1.45–41.58	0.02
The firth's logistic regression model was used to evaluate the risk of mortality. SUA: Single umbilical artery			

## 4. Discussion 

In this study, we observed that cardiac anomalies most frequently co-occurred with SUA, and that neonates with SUA and concurrent gastrointestinal anomalies had poor perinatal outcomes, including a high incidence of initial positive ventilation and intubation, inotropic drug use, and low Apgar scores at 1 and 5 min. We also found that low birth weight, maternal multiparity, gastrointestinal anomaly, and initial cardiac resuscitation were the risk factors for mortality in neonates with SUA.

Since the first description of SUA in 1955 (15), several reports have described its pathogenesis. Although the pathogenesis of SUA remains uncertain, the most widely accepted causes are primary agenesis or atrophy of the umbilical arteries subsequent to thrombosis; accordingly, its incidence increases with gestational age (16, 17). From an embryogenesis point of view, defects in the early steps of differentiation of the mesoderm, such as clonal elimination and hypocellularity during blastogenesis, may also be related to fetal growth restriction, VACTERL association, and SUA (13, 18). For this reason, SUA is strongly associated with VACTERL, and SUA may be the first clue in diagnosing multiple anomalies in prenatal and perinatal periods (19). Some studies have suggested that an SUA should be included as a component of VACTERL association (13). In this study, 5 cases (4.5%) were associated with VACTERL. This was a higher incidence compared to the general incidence of VACTERL association, which is 1 in 10,000–40,000 live births (19).

Previous studies have suggested many risk factors of SUA, including environmental and genetic factors. Its occurrence has been correlated with several maternal factors such as the Caucasian race, older age, multiparity, thalidomide medication or vitamin A overdose during pregnancy, smoking, maternal pregestational diabetes, chronic hypertension, epileptic disease, previous cesarean delivery, and conception using assisted reproductive technologies (12, 20, 21). Moreover, SUA has been found to occur 3–4 times more frequently among twins than among singletons (12, 22). Controversial results regarding sex predominance among individuals with SUA have also been reported. One study reported no difference in the occurrence of SUA between boys and girls (20); however, another study reported a higher incidence of SUA in girls than in boys (2). Furthermore, another study reported that although the incidence of SUA was higher in girls, the prevalence of associated congenital malformations was greater, and the prognosis was worse in boys than in girls (23). Although this study did not analyze the risk of SUA itself, we analyzed the perinatal factors of concurrent congenital anomalies with SUA and we found that neonates with SUA and oligohydramnios had high incidences in cardiac, gastrointestinal, and CNS anomalies.

Chromosomal abnormalities have also been related to SUA, and the incidence of chromosomal abnormalities in fetuses with SUA is reported to be approximately 1.3–15.3% (8). The risk of chromosomal abnormalities can increase from approximately 4% for those with single congenital anomaly to 50% for those with multiple congenital anomalies (24). The most common chromosomal abnormalities were trisomy 18, trisomy 13, and triploidy, which together, accounted for 82.9% of all cases (22, 24). The other abnormalities were trisomy 21 and 16 and monosomy X (20, 22). In our study, 22 neonates underwent chromosomal analysis, and only 4 showed chromosomal abnormalities. One case diagnosed with Edward syndrome (trisomy 18) had a double outlet right ventricle with ventricular septal defect and atrial septal defect.

The mean perinatal mortality among individuals with SUA is approximately 20.0%, with two-thirds of the perinatal deaths being stillborn and one-third being liveborn (23); especially when fetuses with SUA have other congenital abnormalities, the risk of adverse outcomes increases (6, 22). There have also been numerous reports on the incidence and types of associated congenital malformations. Some studies showed that the most common congenital anomalies were the genitourinary (6.48–23.8%) and cardiovascular (6.25–23.8%) anomalies, followed by anomalies of the musculoskeletal system (5.44%) (8, 10, 12, 25). Another study showed that the most common congenital anomalies were cardiovascular, genitourinary, and gastrointestinal anomalies, followed by CNS anomalies (8). Until now, the prognosis of cardiac and renal abnormalities, the most commonly concurrent types of anomalies with SUA, is widely known to be favorable (26, 27). In our study, 74.0% of neonates with SUA had one or more concurrent congenital anomalies, and cardiac anomalies (63.5%) were most commonly associated with SUA, followed by genitourinary (19.2%), gastrointestinal (11.5%), skeletal (11.5%), CNS (4.8%), and facial (4.8%) anomalies. In particular, neonates with SUA and concurrent gastrointestinal anomalies, in which the prognosis has not been well investigated, had a poor perinatal clinical course, including a high incidence of initial positive ventilation and intubation, inotropic drug use, and low Apgar scores at 1 and 5 min. There was also a concurrence of the gastrointestinal anomalies with skeletal anomalies and facial anomalies with CNS and skeletal anomalies. Moreover, we found that a high number of concurrent anomalies was associated with long duration of incubator care, parenteral nutrition, and hospitalization.

Mortality of neonates with SUA was not only related to associated malformations but also to various comorbidities, such as oligohydramnios, polyhydramnios, prolapse of the cord, maternal hypertension, diabetes mellitus, prematurity, low birth weight, and intrauterine fetal growth restriction (5, 28). Furthermore, mortality of neonates with SUA and concurrent multiple malformations is not well elucidated. We attempted to identify the risk factors of mortality in neonates with SUA, although the mortality rate in this study was low. We found that low birth weight, maternal multiparity, gastrointestinal anomaly, and initial cardiac resuscitation were risk factors for mortality in neonates with SUA.

Our study had some limitations. First, there might have been selection bias. Second, the study was conducted retrospectively in a single local hospital in Korea, and the sample size might be too small for the results to be generalized. Third, we could not analyze the risk factors and prognosis of neonates with isolated SUA or those with SUA and a single concurrent congenital anomaly. Fourth, we were not able to attribute the clinical outcomes to individual congenital anomalies because of possible coeffects of other concurrent anomalies. However, our study may have been the first to reveal a relationship between prognosis and the type or number of concurrent congenital anomalies in neonates with SUA.

## 5. Conclusion

Neonates with SUA and concurrent gastrointestinal anomalies, low birth weight, maternal multiparity, and initial cardiac resuscitation had poor outcomes. Therefore, when SUA is detected on prenatal ultrasound, careful antenatal and postnatal surveillance for the fetal growth and detection of concurrent gastrointestinal anomaly are necessary these efforts may reduce adverse perinatal outcomes in neonates with SUA.

##  Data availability

Data supporting the findings of this study are available upon reasonable request from the corresponding author.

##  Author contributions

Lee NH and Choi HJ had full access to all the data in the study and took responsibility for the integrity of the data and the accuracy of the data analysis. Lee NH and Choi HJ performed the conception, design of the study, and the collection and analysis of the data. Choi HJ wrote the first draft of the manuscript. Ethical issues, statistical analysis, and comments to the first and subsequent versions of the manuscript were performed by Lee NH and Choi HJ. All authors read and approved the final manuscript.

##  Acknowledgments

We thank Won Ki Lee (Department of Medical Informatics, School of Medicine, Kyungpook National University, Daegu, Korea) for the statistical analysis. No funding was received to conduct any aspect of this study.

##  Conflict of Interest

The authors declare that there is no conflict of interest.
